# Testing, revision and application of the social anxiety scale for Chinese social media users

**DOI:** 10.3389/fpsyg.2024.1378093

**Published:** 2024-10-09

**Authors:** Yaning Wang, Huan Zhang, Sarenna Bao

**Affiliations:** ^1^School of Journalism and Communication, Northwest University, Xi’an, China; ^2^School of Journalism and Communication, Nankai University, Tianjin, China; ^3^Institute of Communication Studies, Communication University of China, Beijing, China

**Keywords:** social anxiety scale, social anxiety, mobile social media, social media users, young adults, SAS-SMU, scale validation

## Abstract

This study aims to modify the Social Anxiety Scale for Social Media Users (SAS-SMU) to make it more suitable for assessing social anxiety among Chinese social media users, taking into account the unique cultural nuances and social media usage patterns in China. Consequently, a systematic approach was adopted, involving three distinct studies. Study 1 involved translating the English scale into Chinese, conducting interviews with Chinese social media users, and subsequently revising the scale items based on the interview data to ensure cultural appropriateness. Study 2 employed an online survey to collect data and examine the reliability and construct validity of the revised scale, including a two-phase approach: an exploratory factor analysis (EFA) was conducted with 500 participants to identify the underlying factor structure, and a subsequent confirmatory factor analysis (CFA) with 408 participants was used to cross-validate the results. Thus far, this study has developed a social anxiety scale for Chinese mobile social media users (SAS-CMSMU) consisted of 11 items on three factors: Interaction Anxiety, Privacy Concern Anxiety, and Shared Content Anxiety. Study 3 (*N* = 1,006) applied the SAS-CMSMU to assess social anxiety among Wechat users. The results indicated that participants showed a high level of overall social anxiety (M = 3.646 > 3). Specifically, Privacy Anxiety was the most pronounced, followed by Shared Content Anxiety and Interaction Anxiety. Moreover, significant statistical differences in social anxiety levels were found among WeChat users across gender, age, education, income, and relationship status.

## 1 Introduction

Leveraging internet technology, mobile social media platforms offer users immediacy, connectivity, mobility, and affordability, facilitating communication and interaction across time and space. While these platforms have replaced traditional face-to-face interactions, mitigating some communication-related stressors, they have concomitantly introduced new challenges such as social interaction stress and anxiety. A central question in the literature reviewed is the impact of mobile social media on daily life, particularly as it intersects with human social interactions. Research among Generation Z (born in 1996 and later) has indicated that 67% have experienced anxiety, unhappiness, or depression linked to social media use, with 58% seeking relief through platform avoidance (Holliday, 2019). Existing Social Anxiety Scale for Social Media Users (SAS-SMU), while valuable, may not fully capture the unique manifestations of social anxiety in the Chinese context. Factors such as characterized by a strong emphasis on face-saving, interpersonal relationships, and hierarchical structures, particularly in the teacher-student and supervisor-subordinate relationships, as well as the pervasive *“996”*work culture. To address this, the present study develops a scale, adapted from the SAS-SMU, to measure social anxiety among Chinese individuals, specifically within the context of WeChat, a widely used messaging and social media platform.

### 1.1 Social anxiety of mobile social media users

Social anxiety, characterized by the fear of negative evaluation, is often accompanied by feelings of inadequacy, inferiority, self-consciousness, embarrassment, humiliation, and melancholy (Richards, 2013). As a pervasive interpersonal fear, social anxiety primarily manifests in verbal communication contexts. The construct of social anxiety was initially introduced by British psychiatrists Mark and Gelder in 1966. Subsequently, Watson and Friend (1969) defined social-evaluative anxiety as the core component of social anxiety within face-to-face interactions. Schlenker and Leary (1982) further elaborated on this, characterizing social anxiety as emotional distress arising from social situations, interpersonal interactions, and the perception of being scrutinized or judged. In 1983, Mark Leary proposed that social anxiety is an anticipatory response to interpersonal evaluation, a definition that has significantly shaped the field of social anxiety research. Building on Watson and Friend’s earlier work, Leary emphasized the role of social context in eliciting anxiety. Both definitions converge on face-to-face interaction in the development of social anxiety.

The evolution of social anxiety within interpersonal relationships has been influenced by the advent of social media. While some research suggests that social media can serve as a safe space for socially anxious individuals (Pierce, 2009), a growing body of evidence indicates a negative correlation between social media use and mental health outcomes, including anxiety and depression (Grieve et al., 2013; Selfhout et al., 2009; Arikan et al., 2022; Valkenburg et al., 2022; Jia et al., 2022). This research provides a foundation for the present study. While social anxiety traditionally manifests in face-to-face interactions, the advent of social media has necessitated a reconceptualization of the construct. Researchers have sought to define and measure online social anxiety, distinguishing it from its offline counterpart (Chen et al., 2020a; Marino et al., 2023). Notably, Alkis et al. developed the SAS-SMU to assess social anxiety specifically within the context of social media use. It is crucial to emphasize that this instrument is designed to capture the unique anxieties arising from online social interactions.

Social anxiety within the mobile social media context can be conceptualized as a form of “digital stress” arising from the use of digital media and mobile devices. This construct encompasses a range of psychosocial consequences, including subjective emotional, behavioral, and physical experiences (Hefner and Vorderer, 2016; Reinecke et al., 2017), as well as cognitive, affective, and physiological arousal triggered by platform notifications and usage (She and Zhang, 2022; Thomée et al., 2010). Essentially, the stress induced by digital interactions serves as a primary catalyst for social anxiety among mobile social media users (Weinstein and Selman, 2016). Building upon this foundation, we posit that social anxiety within the mobile social media context manifests in both synchronous and asynchronous interactions. This suggests a transition from offline to online social anxiety, compounded by the unique affordances of technology that enable physical detachment (Lin and Jian, 2020). Consequently, social anxiety in the digital realm encompasses a broader spectrum, including anxieties related to direct online interactions (e.g., chatting, content sharing, commenting) and concerns about privacy and information disclosure.

Online social anxiety is a distinct construct from its offline counterpart, representing a maladaptive form of social media engagement (Marino et al., 2023; Chen et al., 2020b). This study focuses on the anxiety experienced by users during social media interactions, emphasizing the psychological distress arising from mediated communication (Marino et al., 2023; Alkis et al., 2017). While research suggests that online interactions can provide a sense of control and reduced anxiety in certain contexts (Fernandez et al., 2012), this study primarily examines the unique stressors arising from the ubilquitous nature of digital media, where individuals are permanently online and permanently connected.

### 1.2 Social anxiety in mediated social interaction: connotations and dimensions

Prior to the advent of social media, conceptualizations of social anxiety significantly influenced the development of measurement scales. Within this paradigm, researchers sought to define and quantify social anxiety, with a particular focus on its underlying dimensions. To examine social anxiety through the lens of social-evaluation anxiety, Watson and Friend (1969) initially proposed two core dimensions: social anxiety avoidance and distress (SAD) and fear of negative evaluation (FNE). Building on this foundation, Leary (1983a) emphasized the negative evaluation component, developing the 12-item Brief Fear of Negative Evaluation Scale (BFNES). Contemporaneously, Leary (1983b) introduced the Interaction Anxiousness Scale (IAS) to assess the affective dimension of social discomfort. Leary and Kowalski (1993) subsequently reaffirmed the construct and criterion-related validity (CRV) of the IAS. Additionally, the Liebowitz Social Anxiety Scale (LSAS) was developed by Heimberg et al. (1999) to evaluate the breadth of social interaction and performance situations inducing fear and avoidance in individuals with social phobia. Collectively, these instruments predominantly focus on assessing the perceived dimensions and intensity of social anxiety within face-to-face contexts.

The integration of social media into everyday life has prompted scholarly attention to the evolving nature of social anxiety. Researchers have increasingly focused on the influence of social media use on this construct. Chen et al. (2020a) characterized online social anxiety as a negative interpersonal experience within the social media context, manifested in Privacy Concerns, Interaction Anxiety, and Fear of Negative Evaluation. Recognizing the shifting dimensions of social anxiety among social media users, Alkis et al. (2017) advocated for the adaptation of both conceptualizations and measurement tools. Through a comprehensive review, they formally introduced the concept and measurement of social anxiety within the social media era. Building on the foundational work of Watson and Friend, Leary, and others, they proposed an operational definition for assessing online social anxiety. Alkis and colleagues further posited that social anxiety among social media users encompasses four primary factors: Shared Content Anxiety, Interaction Anxiety, Privacy Concern Anxiety, and Self-Evaluation Anxiety.

Alkis et al. examined users’ social anxiety within the context of social media activities, building upon existing face-to-face conceptualizations while incorporating novel manifestations of social anxiety in the digital realm. Interaction anxiety and self-evaluation anxiety, established constructs in face-to-face interactions, were reconceptualized to reflect the transition of social engagement from physical to virtual spaces. Conversely, shared content anxiety and privacy concern anxiety emerged as unique phenomena within the mobile social media landscape. Shared content anxiety is engendered by the processes of content creation and dissemination, while privacy concern anxiety represents a negative outcome of new media technologies that offer personalized services through data collection and utilization, particularly regarding concerns over personal information control and misuse.

The inherent portability and mobility of mobile social media have engendered a novel social ecosystem. Portable media devices, such as smartphones, cultivate a mobile media experience characterized by a pervasive sense of “always on and always on them” (Cohen, 2016). While facilitating continuous media consumption, the mobile media environment concurrently subjects users to heightened communication demands (Reinecke et al., 2017). Within the perpetually connected landscape of mobile social media, individuals are immersed in an environment characterized by incessant content availability and online interaction (Vorderer et al., 2016). This condition amplifies users’ susceptibility to novel forms of social anxiety and presents increased opportunities for encountering online social challenges.

The scale developed by Alkis and colleagues, rooted in Turkish social media usage patterns, may not fully capture the nuances of Chinese social media platforms. Given the established influence of cultural factors on social anxiety manifestations (Spence and Rapee, 2016), cross-cultural validation is imperative. Moreover, the SAS-SMU overlooks the impact of continuous connectivity and other technological attributes of mobile media on individuals’ social lives and perceptions of social anxiety. Building upon the work of Alkis et al., this study introduces two critical variables: the distinctive social context of China and the technological characteristics of mobile media. The objective is to assess the applicability of the SAS-SMU to Chinese mobile social media users and to refine it into a scale suitable for measuring social anxiety within the unique context of Chinese mobile social interaction.

In summary, this study employs WeChat as a representative platform for investigating mobile social media within the Chinese cultural context. Building upon the SAS-SMU, the research develops a scale tailored to assessing social anxiety among Chinese mobile social media users. Subsequently, the scale is applied to measure the extent of social anxiety experienced by WeChat users during platform interactions. This study aims to address the following research questions:

Q1: Which items within the SAS-SMU are culturally appropriate and which are culturally inappropriate for the Chinese social context?

Q2: What modifications are necessary to develop a culturally adapted SAS-SMU that is sensitive to the mobile social media users in China?

Q3: What are the specific manifestations of social anxiety in the context of WeChat use among Chinese users?

## 2 Study overview

This multi-study investigation hypothesizes a process for adapting and validating the SAS-SMU for Chinese mobile social media users. This process involves translation, exploratory study, scale refinement, and reliability and validity testing (EFA and CFA), followed by the application of the revised scale. To achieve this goal, three studies were conducted sequentially. Study 1 involved the translation of Alkis et al.’s SAS-SMU into Chinese. Two graduate students were invited to review the translation. Then, a pilot study with 17 mobile social media users was conducted to assess the comprehensibility and relevance of the translated scale. Based on the results of in-depth interviews, preliminary revisions were made to the Chinese version of the SAS-SMU. Study 2 aimed to refine the Chinese version of the SAS-SMU based on the findings from Study 1. First, exploratory factor analysis (EFA) was conducted on data from 500 Chinese social media users to identify the underlying factor structure of the scale. Second, confirmatory factor analysis (CFA) was conducted on a separate sample of 408 participants to validate the factor structure obtained in the EFA. Study 3 employed the revised SAS-SMU to measure social anxiety in a sample of 1,006 Chinese social media users.

## 3 Study 1: the translation and construction of the Chinese version of the SAS-SMU

### 3.1 Method

#### 3.1.1 Procedure

The SAS-SMU is a 21-item Likert scale anchored on a five-point response format. The primary objective of this study is to develop a culturally adapted Chinese version of the SAS-SMU. The translation process will involve a forward-backward translation procedure, which was completed jointly by professional translators and research objects. The translators’ work ensured the accuracy of the translated statements into Chinese, and subsequent interviews with research participants guaranteed the cultural appropriateness of the translated statements within the Chinese context. Particular attention will be paid to modifying items to reflect the unique social anxiety-inducing situations commonly encountered by Chinese individuals. Additionally, new items will be added to capture specific social anxiety scenarios frequently reported by participants. The following section outlines the specific procedures undertaken to develop this scale.

Step 1: We conducted a forward translation of the English scale into Chinese. This step retains the four-factor structure and 21-item SAS-SMU.

Step 2: Two graduate students majoring in English were independently tasked with proofreading the initial translation, resulting in the final Chinese version of the SAS-SMU.

Step 3: In-depth interviews were conducted to assess the usability and refine the Chinese version of the SAS-SMU. Participants were presented with the scale items and asked to evaluate their clarity. Additionally, they were queried about the frequency and relevance of the described social media scenarios. Finally, participants were prompted to identify any additional anxiety-inducing situations related to social media that were not captured by the scale.

#### 3.1.2 Participants

To explore the SAS-SMU and conduct initial item refinement, a convenience sample of 17 active young mobile social media users was selected for in-depth interviews. Data collection methods included face-to-face interviews, voice calls, and written responses (Table 1).

**Table 1:** List of in-depth interviewers conducted during the initial scale construction.

NO.	Gender	Age	Occupation	Residence	Interview way
SF1	Female	26	Website editor	Xi’an, Shanxi	WeChat
SF2	Female	28	Journal editor	Beijing	Face to face
SF3	Female	28	Event planner in CMG Mobile	Beijing	Face to face
SF4	Female	29	Internet marketing agent	Beijing	Face to face
SF5	Male	29	Programmer	Beijing	Face to face
SF6	Female	30	High school teacher	Baoji, Shanxi	WeChat
SF7	Female	30	Online teacher	Xi’an, Shanxi	WeChat
SF8	Female	30	News editor	Beijing	Face to face
SF9	Male	29	Aerospace engineer	Beijing	WeChat
SF10	Male	29	PhD student	Beijing	Face to face
SF11	Male	28	PhD student	Beijing	Face to face
SF12	Male	32	PhD student	Beijing	Face to face
SF13	Female	24	PhD student	Beijing	Face to face
SF14	Male	32	Civil servant	Beijing	Written reply
SF15	Female	29	Event planner in real estate industry	Shanghai	WeChat
SF16	Male	35	HR manager	Beijing	Face to face
SF17	Female	28	Bank clerk	Xining, Qinghai	WeChat

### 3.2 Results

In-depth interviews were conducted with Chinese mobile social media users to assess the cultural appropriateness of the SAS-SMU measurement items. Through thematic analysis guided by the principle of theoretical saturation, The scale revision yielded four results. First, statements that resonated with participants’ social anxiety experiences were retained. Second, statements that were not entirely suitable for the Chinese context were adjusted. Third, statements frequently mentioned by respondents but not included in the original scale were added. Fourth, statements were entirely unsuitable for the Chinese context were removed. Finally, six statements within the SAS-SMU underwent modification during the initial phase of the online social anxiety study: one statement was eliminated, one was introduced, and four were revised. These changes were informed by the majority opinion of participants, as indicated by frequent suggestions to modify or remove specific items during in-depth interviews. The detailed findings of the revision are presented below:

One statement was eliminated based on the Chinese context. Specifically, the statements “*I am afraid that my close friends will not approve of my behavior*” and “*I am concerned about disapproval of my behaviors by others*” have a high degree of similarity. Since close friends are included in the category of others in specific group divisions, respondents indicated that these statements were synonymous and could lead to misunderstandings in survey responses. Additionally, all respondents gave a score of 1 (not conforming) to S4. They pointed out that if the content shared is with close friends, there is generally no situation where friends disapprove of the behavior (nine respondents expressed this view and the remaining eight were asked for their opinion on this point, and they agreed with it). Someone who participated in the interview stated plainly, “*I will not have such a person among my good friends, and if I do, then he or she will be removed from my circle of friends*” (SF2). Therefore, the item S4 was eliminated.

One new statement was introduced into the scale based on the feedback from the participants. Item I7 was developed through an integration of the Interaction Anxiety Scale (IAS; Leary, 1983b) and qualitative findings. The IAS includes the item “*I get nervous when I must talk to a teacher or boss,*” a scenario that, based on researchers’ observation, persists in online environments. In-depth interviews confirmed this notion, with participants reporting anxiety in interactions with authority figures on social media platforms. To capture this dimension, an additional item was included: “Even when communicating with leaders or teachers via instant messaging, I still feel nervous.” This item was unanimously endorsed by participants.

Four statements underwent modification, namely S2, P4, I3, I4. The adjustments are as follows: Item S2 was modified from *“I am worried that the content I share will be ridiculed by others”* to “*I am worried that the content I share will cause misunderstanding among others.”* While more than half of participants reported not experiencing direct ridicule for their posts, a significant proportion expressed concern about content being misinterpreted by unfamiliar individuals. Such as participant SF4 said, *Sometimes I post something in WeChat moments just to express the state of mind at the time, but will be misunderstood by some people, really made me very speechless.*

The item P4 “*I would be concerned if my personal space is accessed without my consent*” was adjusted to “*I am reluctant to let unfamiliar people access my personal space or WeChat Moments*.” Chinese social media platforms typically provide users with granular control over the visibility of their posts and personal information. Features such as friend verification, post visibility settings (e.g., three days, one month, indefinitely), and limits on the number of public posts often mitigate concerns about unauthorized access. The following are quotes directly from the respondents, “*I do not worry about it at all because I can set it up myself. I can show it to whoever I want, and how long they can see if I want.”* (SF7) “*This does not worry me. I can decide what to let my friends see by grouping them and setting the length of time for content to be shown. But I do not like people I am not familiar to see my posts”* (SF12).

Items I3 and I4 underwent similar modifications due to the distinct characteristics of online interaction contexts. Items originally designed for face-to-face interactions were adapted to better assess social anxiety within the online environment.

The item I3 “*feel uneasy while making new friends”* was adjusted “*I feel worried when I received a friend request from strangers on social media.”* This adjustment reflects the unique dynamics of online social interactions, where friend acquisition often initiates with a friend request.

Item I4 was modified from *“I feel tense when I meet someone for the first time”* to *“I feel nervous when I chat with a new friend on social app for the first time by voice or video.”* This adjustment aligns with the unique characteristics of online social interaction. While text-based communication on social media can mitigate social anxiety due to asynchronous interaction, as suggested by Peter and Valkenburg (2006), real-time modalities such as voice and video calls demand more immediate social presence, akin to face-to-face encounters (see Table 1).

As shown in Table 2, this study has successfully constructed a social anxiety scale tailored for the specific needs of this research. The “*revised*” designation in the *Notes* column signifies that the original wording has been modified according to the results of the exploratory research phase’s in-depth interviewees. The “*added*” designation in the *Notes* column signifies statements that have been newly introduced and were absent from the original scale. The “*reserved*” designation in the *Notes* column signifies that the original sentences from the scale have been retained, with only a translation performed. Items labeled “*new*” denote modifications made to tailor the scale to the Chinese context.

**Table 2:** Translation of the English version of SAS-SMU.

Factor	Items		Items in Chinese	Notes
Shared content anxiety	S1	I feel anxious about the fact that others might find my actions awkward.	我担心自己在社交媒体上的一些做法让让他人感到别扭	Reserved
	S2	I am concerned about being ridiculed by others for the content I have shared.	我担心自己分享的内容被别人嘲笑	Adjusted to S2new
	S2new	—	我担心自己分享的内容让别人产生误会(I am worried that the content I share will cause misunderstanding)	
	S3	I am concerned about the fact that the content I share will not be liked by others.	我担心别人不喜欢我分享的内容	Reserved
	S4	I am afraid that my close friends will not approve of my behavior.	我害怕好朋友不赞成我在社交媒体上的形式方式	Removed
	S5	I would feel uncomfortable when my friends publicly express their dislike about content I have shared.	当朋友剬开表达他们不喜欢我分享的内容时, 我会感到不舒服	Reserved
	S6	I am concerned about disapproval of my behaviors by others.	我担心别人不赞成我在社交APP上的行事方式	Reserved
	S7	I am concerned about being judged about my shared content by my friends in the presence of others.	我在意朋友对我分享的内容的评价	Reserved
Privacy concern anxiety	P1	The possibility of having my private information acquired by others makes me feel anxious.	想到别人有可能会获取我的私人信息, 我会感到担忧	Reserved
	P2	The possibility of having my private information shared publicly makes me anxious.	意识到我的私人信息有可能被剬开, 会让我担心	Reserved
	P3	I feel uneasy when my friends share my private information with people I do not know.	当朋友和我不认识的人分享我的私人信息时, 我感到不安	Reserved
	P4	I would be concerned if my personal space is accessed without my consent.	如果我的个人空间未经本人同意被访问, 我会感到担忧	Adjusted to P4new
	P4new	—	我不愿意让不熟悉的人访问我的个人空间或朋友圈(I am reluctant to let unfamiliar people access my personal space or WeChat Moments)	
	P5	I feel anxious about how social media companies/executives handle privacy policy regarding my private life.	我对社交媒体平台如何处理有关我私人生活的隐私政策感到担忧	Reserved
Interaction anxiety	I1	I feel anxious when talking with people I have just met.	和刚刚认识的人聊天我会感到紧张	Reserved
	I2	I feel nervous when I talk with people I do not know very well.	和不太熟悉的人聊天我会感到不自在	Reserved
	I3	I feel uneasy while making new friends.	结识新好友会让我感到担忧	Adjusted to I3new
	I3new	—	当不认识的人添加我为好友时, 我会感到担忧(I feel worried when I received a friend request from strangers on social media)	
	I4	I feel tense when I meet someone for the first time.	第一次与别人聊天让我感到紧张	Adjusted to I4new
	I4new	—	第一次和社交APP上新加的好友语音或视频聊天, 让我感到紧张(I feel nervous when I chat with a new friend on social app for the first time by voice or video)	
	I5	I am afraid of interacting with others.	我不擅长和别人打交道	Reserved
	I6	I feel nervous when I have to talk with others about myself.	当不得不和别人谈论我自己时, 我感到紧张	Reserved
	I7	—	即使通过手机与领导/老师交流, 我还是会感到紧张(I feel nervous even if I communicate with leaders/teachers via cellphone)	Added
Self-evaluation anxiety	SE1	I feel anxious about making a negative impression on people.	我会因为给别人留下不好的印象而感到焦虑	Reserved
	SE2	I am concerned about people thinking poorly of me.	我担心他人给我的评价不好	Reserved
	SE3	I feel anxious about not being able to meet people’s expectations	我会因为不能满足别人的期望而觉得沮丧	Reserved

## 4 Study 2: testing the reliability and validity of the new scale

Following the preceding work, a preliminary version of the Social Anxiety Scale has been developed. Subsequent analyses will focus on establishing the scale’s reliability and validity.

### 4.1 Method

#### 4.1.1 Procedure

Study 2 involved a two-phase data collection process to refine the structure of the SAS-SMU in Chinese context. Both phases employed convenience sampling to recruit participants. Exploratory factor analysis (EFA) was conducted on the first dataset to identify the underlying factor structure of the scale. The second dataset was used to cross-validate the results obtained from the EFA.

In data collection, Participants were recruited through an online survey disseminated via the Wenjuanxing platform among WeChat users. A snowball sampling method was adopted to expand the sample size. To ensure data quality, responses with unusually short completion times (under 50–60 s) were excluded. The social anxiety scale employed a 5-point Likert format to assess respondent agreement with the described social anxiety scenarios (1 = strongly disagree, 2 = disagree, 3 = neutral, 4 = agree, 5 = strongly agree). The collected data was subjected to both EFA and CFA to develop and validate a social anxiety scale tailored for Chinese individuals engaging in mobile social media. The refined scale is capable of accurately measuring the level of social anxiety in this specific population.

#### 4.1.2 Participants

A total of 500 valid responses were collected for the EFA phase, with a predominantly female sample (*n* = 345, 69%). Participants ranged in age from 18 to 55 years, with a majority (84.4%) aged 18–35 and a minority (15.6%) aged 36–55. The sample was highly educated, with 90% holding at least a bachelor’s degree. For the CFA phase, 408 valid responses were obtained, including 252 females (61.8%). 84.3% of them were aged 18–35. Data collection and utilization adhered strictly to ethical guidelines, with informed consent obtained from all participants. Based on the collected data, a preliminary five-point Likert scale assessing social anxiety among Chinese mobile social media users was developed.

#### 4.1.3 Data analysis

Data analysis for scale refinement was conducted in three phases. Firstly, exploratory factor analysis (EFA) was employed to identify the underlying factor structure of the scale. Secondly, confirmatory factor analysis (CFA) was conducted to validate the factor structure obtained from the EFA. Finally, the internal consistency reliability of each factor was assessed using Cronbach’s alpha.

### 4.2 Exploratory factor analysis

A preliminary survey was administered to gather feedback on questionnaire design, item clarity, and to identify potential invalid questions. Data from this pre-survey informed the scale’s refinement process, including reliability and validity testing. SPSS 26 was employed to conduct these analyses. The following steps were taken to conduct the EFA.

An initial internal consistency analysis of the 20-item scale yielded a Cronbach’s alpha of 0.923. Item-total statistics indicated that removing item P4new (*I am reluctant to let unfamiliar people access my personal space or WeChat Moments*) could enhance scale reliability. Subsequent analysis of the 19-item scale resulted in a slightly improved Cronbach’s alpha of 0.924.

An exploratory factor analysis (EFA) was conducted on the 19-item scale using principal component analysis with varimax rotation, retaining factors according to the scree plot and eigenvalues greater than 1. The Kaiser-Meyer-Olkin (KMO) measure of sampling adequacy was 0.911, indicating a suitable sample for factor analysis. Bartlett’s test of sphericity was highly significant (χ^2^ = 6308.880, df = 171, *p* < 0.001), further supporting the factorability of the data.

The initial EFA yielded a four-factor scale. However, A second EFA was appliyed to the 19 items that showed two items demonstrated cross-loading issues and then they were subsequently removed. The retained three factors were interpreted as relating to social anxiety arising from interaction, social anxiety arising from shared content, and social anxiety arising from privacy concern, as detailed in the Table 3.

**Table 3:** Results of EFA for social anxiety.

		Rotated component matrix^a^			
			Factor		
			1	2	3
IA	I1	I feel nervous when I talk with people I do not know very well.	0.821		
	I2	I feel nervous when I have to talk with others about myself.	0.776		
	I3	I am afraid of interacting with others.	0.775		
	I4	I feel anxious when talking with people I have just met.	0.773		
	I5	I feel nervous when I talk to a new friend on social app for the first time by voice or video.	0.695		
	I6	I feel worried when strangers add me as a friend on social media.	0.663		
	I7	I feel nervous even if I communicate with leaders/teachers via cell phone.	0.454		
SCA	S1	I am concerned about disapproval of my behaviors by others.		0.822	
	S2	I am worried that the content I share will cause misunderstanding.		0.810	
	S3	I am concerned about being judged about my shared content by my friends in the presence of others.		0.808	
	S4	I would feel uncomfortable when my friends publicly express their dislike about content I have shared.		0.793	
	S5	I feel anxious about the fact that others might find my actions awkward.		0.692	
	S6	I feel anxious about making a negative impression on people.		0.611	
PCA	P1	The possibility of having my private information shared publicly makes me anxious			0.884
	P2	The possibility of having my private information acquired by others makes me feel anxious.			0.858
	P3	I feel anxious about how social media companies/executives handle privacy policy regarding my private life.			0.798
	P4	I feel uneasy when my friends share my private information with people I do not know.			0.760

^a^Rotation converged in 5 iterations.

The Kaiser-Meyer-Olkin (KMO) measure of sampling adequacy was 0.897, and Bartlett’s test of sphericity was highly significant (χ^2^ = 5199.462, df = 136, *p* < 0.001). In this study, the three-factor model accounts for 65.19% of the cumulative percentage of variation explained by retained factors and each factor has a minimum of three items.

Based on the dimensional structure of the SAS-SMU scale, three distinct factors underlying social anxiety related to mobile social media were achieved: Interaction Anxiety (IA), Shared Content Anxiety (SCA), and Privacy Concern Anxiety (PCA).

### 4.3 Confirmatory factor analysis

To confirm the factor structure derived from the exploratory factor analysis (EFA), a confirmatory factor analysis (CFA) was conducted using SPSS Amos 25. The CFA aimed to assess the congruence between the hypothesized factor model and the observed data.

A CFA was conducted using AMOS to assess the hypothesized factor structure. Items were assigned to their respective latent factors based on the EFA results. Maximum likelihood estimation was employed to estimate model parameters. Fit indices revealed a suboptimal model fit, as indicated by the following values: χ^2^/df = 3.850, GFI =0.892, AGFI =0.858, CFI = 0.925, and RMSEA =0.084. While the χ^2^/df and CFI values approached acceptable thresholds, the overall fit indices suggest the need for model refinement.

Model modification was guided by two criteria: (a) standardized factor loadings below 0.6, indicating an item’s weak association with its factor, (b) the modification indices (MIs) generated by AMOS, prioritizing the removal of items with larger MIs when multiple items met the first criterion. These criteria were applied iteratively until model fit was optimized.

The initial model demonstrated adequate fit, with all standardized factor loadings exceeding 0.6. However, subsequent model modifications were necessitated based on MIs. Deletion of item I4 resulted in a revised model with marginal fit, as indicated by AGFI and SRMR values of 0.889 and 0.0621, respectively. Given the suboptimal fit, further model refinement is required.

**Figure 1:**
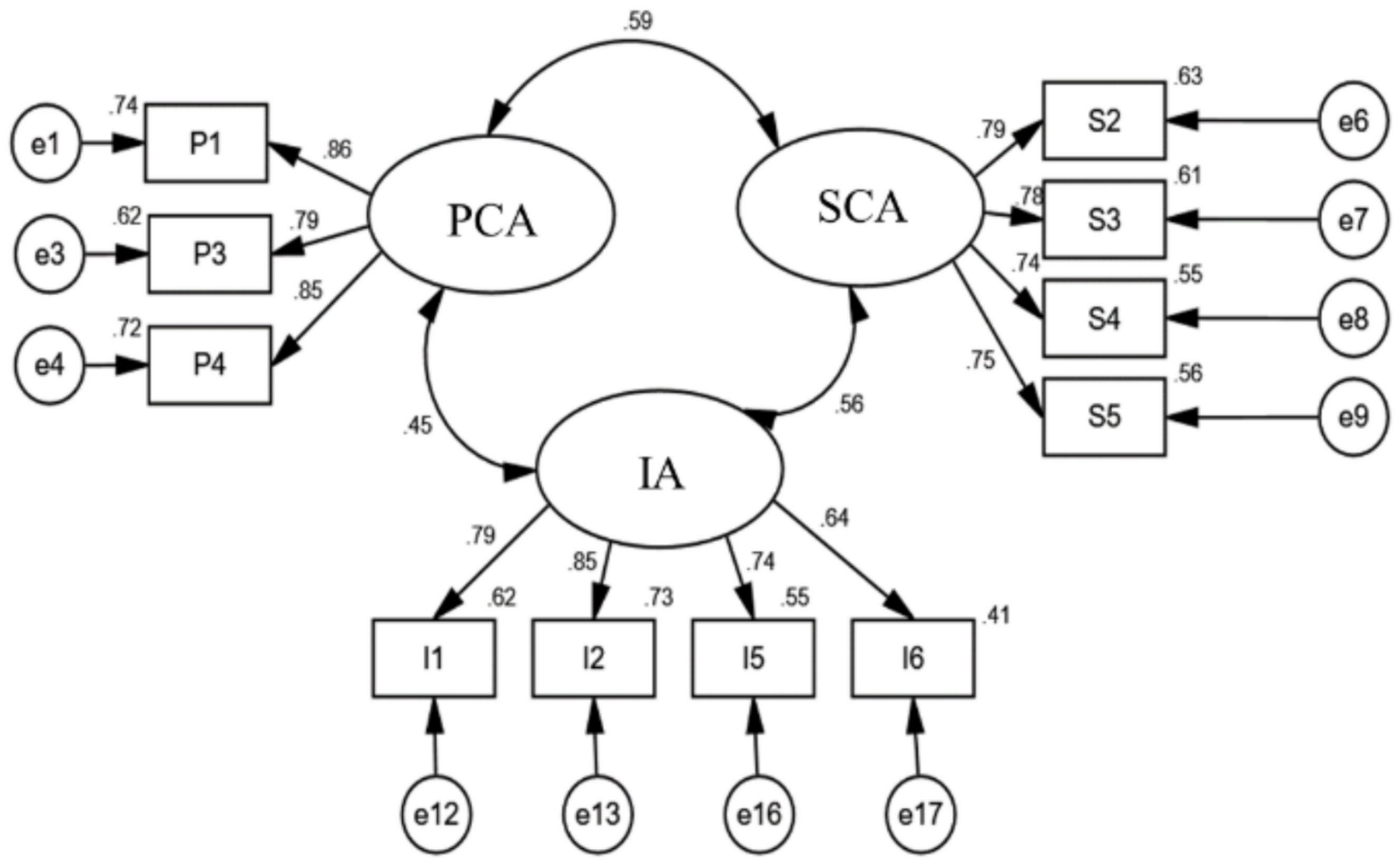
Standardized coefficients for three-factor model the social anxiety scale for Chinese mobile social media users.

Goodness-of-fit indices were compared to established cut-off values for each model revision until an acceptable fit was attained. Through iterative model modifications, involving the removal of items P2, I3, I4, I7, S1, and S6, the final model achieved satisfactory fit indices: χ^2^/df = 2.636, GFI = 0.979, AGFI = 0.968, CFI = 0.987, SRMR = 0.038, and RMSEA = 0.044 (Table 4). Figure 1 presents the standardized factor loadings for the three social anxiety factors. All standardized factor loadings exceeded 0.6, indicating strong relationships between items and their respective factors, thus demonstrating the scale’s capacity to effectively measure the underlying dimensions.

**Table 4:** Goodness-of-fit indices of three-factor model for the social anxiety scale for Chinese mobile social media users.

Indicators	χ^2^/df	GFI	AGFI	CFI	SRMR	RMSEA
Fitting indicator	2.636	0.979	0.968	0.987	0.038	0.044
Ideal indicator	<5	>0.9	>0.9	>0.9	<0.05	<0.08

Subsequently, the convergent validity of the factors was assessed. To evaluate the internal consistency of the measurement items within each factor of the CFA model, item reliability was examined. This analysis determined the extent to which the items accurately reflected the underlying latent constructs.

Convergent validity, a key facet of measurement model assessment, is typically evaluated through two established metrics: average variance extracted (AVE) and composite reliability (CR) (Carmines and Mciver, 1981). AVE reflects the degree to which the variance in a construct’s reflective indicators is attributable to the underlying latent variable. An AVE value exceeding 0.5 is generally considered indicative of acceptable convergent validity, signifying that a majority of the variance in the measures reflects the intended construct (Fornell and Larcker, 1981; Hair et al., 2009). Conversely, CR estimates the internal consistency of a construct by assessing the extent to which its indicators collectively capture the latent variable (Bagozzi and Yi, 1988). A CR value greater than or equal to 0.7 suggests that the indicators demonstrate sufficient reliability in measuring the construct (Hair et al., 2009). As presented in Table 5, the AVE and CR values for all constructs surpass the recommended thresholds, providing evidence of satisfactory convergent validity for the measurement model.

**Table 5:** Convergent validity of the social anxiety factors.

Factor	Item	Unstd.	S.E.	*t*	*P*	Std.	SMC	AVE	CR
PCA	P1	1.000				0.859	0.738	0.693	0.871
	P3	0.879	0.049	17.816	***	0.786	0.618		
	P4	1.015	0.053	19.324	***	0.850	0.722		
SCA	S2	1.000				0.792	0.627	0.586	0.850
	S3	0.999	0.063	15.916	***	0.783	0.613		
	S4	0.949	0.063	14.953	***	0.739	0.546		
	S5	0.995	0.066	15.159	***	0.748	0.560		
IA	I1	1.000				0.790	0.624	0.577	0.844
	I2	1.076	0.063	17.154	***	0.855	0.731		
	I5	0.953	0.064	15.001	***	0.739	0.546		
	I6	0.797	0.063	12.746	***	0.639	0.408		

To complement the assessment of convergent validity, discriminant validity analysis was conducted to ascertain the distinctiveness of the latent constructs. This analysis aimed to determine whether the proposed factors represented empirically distinct dimensions. According to Fornell and Larcker (1981), a factor exhibits discriminant validity if the square root of its AVE is larger than the highest correlation coefficient shared with any other factor. This criterion ensures that the internal consistency within a construct surpasses its external relationships with other constructs.

As indicated in Table 6, the square roots of the AVEs for IA, SCA, and PCA are 0.760, 0.766, and 0.832, respectively. Each of these values exceeds the absolute value of the corresponding correlation coefficients between the respective factor and the remaining two factors. This pattern provides empirical support for the discriminant validity of the constructs, as the internal consistency of each factor is more pronounced than its shared variance with other factors.

**Table 6:** Discriminate validity of social anxiety factors.

	AVE	IA	SCA	PCA
IA	0.577	**0.760**		
SCA	0.586	0.564	**0.766**	
PCA	0.693	0.447	0.595	**0.832**

Bold values in the square roots of the AVE values of these factors.

### 4.4 Reliability analysis with the second sample

The internal consistency of the Social Anxiety Scale was assessed through Cronbach’s alpha coefficient and item-total correlations. The alpha coefficients for the IA, PCA, and SCA subscales were 0.839, 0.870, and 0.849, respectively, indicating robust internal consistency for each dimension. Furthermore, item-total correlations within each subscale exceeded 0.59, providing additional evidence of the scale’s homogeneity.

## 5 Study 3: application of the scale for Chinese mobile social media users

The Social Anxiety Scale for Chinese Mobile Social Media Users (SAS-CMSMU) underwent further refinement and validation in Study 2. The third phase of the study employed the revised SAS-CMSMU to assess the social anxiety levels of WeChat users.

### 5.1 Method

#### 5.1.1 Procedure and measures

In Study 3, the revised Social Anxiety Scale for Mobile Social Media Users (SAS-SMU) was administered to a sample of Chinese WeChat users to assess their levels of social anxiety. Furthermore, we examined the association between social anxiety and various demographic characteristics among this population. Data were collected using an online questionnaire, resulting in a sample of 1,223 respondents. After data cleaning, a final sample of 1,006 participants was retained for analysis. Statistical analyses were conducted to explore the mean levels of social anxiety and to examine how the differences in social anxiety across various demographic groups.

#### 5.1.2 Participants

The final sample of 1,006 participants was predominantly female (53.2%) and well-educated, with 85.4% holding at least a bachelor’s degree. In terms of occupation, 24.1% were full-time students, 25.3% were employed by companies or enterprises, and 19.3% were internet practitioners. Additional demographic information, including residence, relationship status, and income, is presented in Table 7.

**Table 7:** Demographic characteristics of survey respondents (*N* = 1,006).

		Frequency	Percentage			Frequency	Percentage
Gender	Male	535	53.2	Occupation	Full-time student	242	24.1
	Female	471	46.8		Civil servant	163	16.2
Age	18–25	448	44.5		Culture and media Practitioners	80	8.0
	26–30	307	30.5		Internet Practitioners	194	19.3
	31–35	137	13.6		Company or enterprise Employees	255	25.3
	36–45	76	7.6		Freelance	33	3.3
	46–55	38	3.8		Self-employed	9	0.9
Educational Background	High school or below	50	5.0		Housewife	4	0.4
	college degree	97	9.6		laid-off, Unemployment	10	1.0
	Bachelor’s degree	580	57.7		Others	16	1.6
	Master’s degree or above	279	27.7	Income (¥)	1,000	137	13.6
Relationship Status	single	486	48.3		1,000–3,000	151	15.0
	Being in love	250	24.9		3,001–5,000	195	19.4
	Married with no kids	83	8.3		5,001–8,000	230	22.9
	Married with Kids	182	18.1		8,001–10,000	118	11.7
	Single with Kids	5	0.5		10,001–20,000	123	12.2
Habitual residence	Metropolis	343	34.1		≥20,001	52	5.2
	New first- tire cities	362	36.0				
	Second tier cities	184	18.3				
	Other cities	100	9.9				
	countryside	17	1.7				

#### 5.1.3 Data analysis

In application of revised scale, an independent sample t-test were conducted to examine the levels of overall social anxiety and anxiety on three dimensions of the revised scale among the participants. Furthermore, we conducted analysis of variance (ANOVA) to further examine how the overall level of social anxiety change based on different sociodemographic variables. Specifically, an independent samples t-test was applied to analyze gender differences, while an F-test was utilized to examine the variance in social anxiety across different age groups, levels of education, income brackets, and relationship statuses, thereby assessing whether social anxiety varies significantly among these sociodemographic groups. Subsequently, we employed the revised scale to measure the level of social anxiety among Chinese social media users on WeChat (see Table 8).

**Table 8:** The level of each sub-dimension of social anxiety and the overall status of social anxiety (*N* = 1,006).

	M	SD	Inspection value = 3				
			*t*	Sig.(two-sided)	Mean difference	Difference 95% confidence interval	
						Lower limit	Upper limit
Shared content anxiety	3.664	0.887	23.750	0.000	0.664	0.609	0.719
Privacy concern anxiety	3.942	0.903	33.090	0.000	0.942	0.886	0.998
Interaction anxiety	3.406	1.000	12.886	0.000	0.406	0.344	0.468
**SAS-CMSMU**	3.646	0.763	26.845	0.000	0.646	0.599	0.693

### 5.2 Results

The psychometric properties of the SAS-CMSMU were examined through Cronbach’s alpha and confirmatory factor analysis (CFA). The scale demonstrated adequate reliability (*α* = 0.896) and convergent validity, as indicated by acceptable fit indices in the CFA (χ^2^/df = 2.543, GFI = 0.982, AGFI = 0.971, CFI = 0.989, SRMR = 0.0245, RMSEA = 0.039). A one-sample t-test revealed significantly higher mean levels of social anxiety than the Likert scale midpoint (M = 3) across all scale dimensions. Privacy Concerned Anxiety emerged as the most prominent subscale (M = 3.942), followed by Shared Content Anxiety (M = 3.664) and Interact Anxiety (M = 3.406), suggesting a substantial prevalence of social anxiety among participants.

Sociodemographic factors significantly influenced overall social anxiety levels. Gender, age, education, income, and relationship status demonstrated significant associations with social anxiety. As indicated in Table 9, males reported significantly higher levels of social anxiety compared to females (t = 2.12, *p* = 0.035, M_males_ = 3.694, M_females_ = 3.592). Educational attainment exhibited a positive correlation with social anxiety, with individuals possessing a bachelor’s degree reporting the highest levels (M = 3.731). A curvilinear relationship was observed between income and social anxiety, with those earning between 8,001 and 10,000 yuan experiencing the highest levels (M = 3.859). Relationship status significantly influenced social anxiety levels, with distinct patterns emerging among different groups. Single parents reported the lowest levels of social anxiety, with a mean score of 2.964, possibly attributed to the demanding responsibilities of single parenthood and a reduced opportunity for introspection. In contrast, unmarried individuals, particularly those engaged in romantic relationships, showed the highest levels of social anxiety (M = 3.800). This elevated anxiety among this group may be attributed to their heightened emotional sensitivity and a greater focus on interpersonal dynamics, which could lead to increased anxiety levels.

**Table 9:** Differences in total levels of interaction anxiety by demographic variables (*N* = 1,006).

Item	Category	M	SD	T/F	*P*
Gender	Male	3.694	0.758	2.116	**0.035**
	Female	3.592	0.766		
Age	18–25	3.679	0.707	4.834	**0.001**
	26–30	3.736	0.749		
	31–35	3.583	0.821		
	36–45	3.422	0.794		
	46–55	3.201	0.994		
Educational background	High school or below	3.489	0.889	7.473	**0.000**
	College degree	3.679	0.744		
	Bachelor’s degree	3.731	0.734		
	Master’s degree or above	3.485	0.779		
Income (¥)	≤1,000	3.607	0.709	8.722	**0.000**
	1,000–3,000	3.696	0.730		
	3,001–5,000	3.602	0.796		
	5,001–8,000	3.721	0.753		
	8,001–10,000	3.859	0.620		
	10,001–20,000	3.625	0.803		
	≥20,001	3.003	0.785		
Relationship status	Single	3.664	0.723	6.486	**0.001**
	Being in love	3.800	0.684		
	Married with no kids	3.566	0.739		
	Married with Kids	3.442	0.917		
	Single with Kids	2.964	0.661		

Bold values in represents the significance test of difference results of users with different demographic characteristics on the level of social anxiety on mobile social media.

## 6 Discussion and conclusion

### 6.1 Testing and revision of SAS-SMU: social anxiety scale for mobile social media users in China

Since its development by Alkis et al. (2017), the Social Anxiety Scale for Social Media Users (SAS-SMU) has been widely adopted to investigate the relationship between social media use and psychological well-being. Research has spanned diverse populations and contexts, with applications ranging from examining social anxiety among Chinese WeChat users (Wang and Zhang, 2020; Wang, 2010) to exploring the association between social anxiety and overall social media use (Yin et al., 2022). Moreover, specific subscales of the SAS-SMU, such as Privacy Concern Anxiety and Self-evaluation Anxiety, have been employed to elucidate underlying mechanisms, as evidenced by studies examining the mediating role of affordance in the relationship between social anxiety and online safety-seeking behavior. While the Social Anxiety Scale for Social Media Users has gained traction in research, psychometric evaluations, particularly in cross-cultural settings, remain relatively scarce. Existing studies, such as those by Jia et al. (2022), Erliksson et al. (2020), Liu and Ma (2020), and Wang (2021), have highlighted the need for further investigations into the scale’s underlying factor structure, internal consistency, and construct validity across diverse cultural contexts. This research gap serves as the primary motivation for the present study. In contrast to previous research (e.g., Alkis et al., 2017), the current study identified a three-factor structure for the SAS-SMU. While these findings align with our conceptualization of the construct, the specific factor composition diverges from prior work.

Several studies have explored the factor structure of the Social Anxiety Scale for Social Media Users. Chen et al. (2020b) identified a three-factor structure for the Chinese version, comprising Evaluation Anxiety, Interaction Anxiety, and Privacy Concern Anxiety. Erliksson et al. (2020) proposed a similar structure, albeit with different factor labels. Notably, Jia et al. (2022) also reported a three-factor structure for the Chinese SAS-SMU, including Social Recognition Anxiety, Interaction Anxiety, and Privacy Concern Anxiety. While consistent in terms of the number of factors, variations in factor composition across these studies suggest the need for further refinement of the factor structure of SAS-SMU, particularly with regard to the conceptualization of shared content anxiety and self-evaluation anxiety. This study extended prior research by examining the SAS-SMU within the Chinese context, considering the evolution of media platforms from Web 2.0 to Web 3.0. To accommodate these technological advancements, the scale underwent rigorous refinement, yielding a three-factor structure of 11-item scale. Notably, one item previously categorized as self-evaluation anxiety was reclassified as shared content anxiety due to conceptual alignment. This modification, while deviating from the original scale, converges with findings from other cross-cultural adaptations of the SAS-SMU. The adapted scale demonstrated robust psychometric properties, including internal consistency reliability, supporting its validity and reliability for assessing social anxiety among Chinese mobile social media users.

In addition to the aforementioned, there have been innovative findings in the validation of the Social Anxiety Scale for Social Media Users (SAS-SMU). Raimondi et al. (2024) conducted a study on the psychometric properties of the SAS-SMU and concluded that the original four-factor model is more appropriate for the Italian general population. In contrast, the competing model proposed by Erliksson et al. (2020), which suggests a three-factor structure for the SAS-SMU, did not fit the data in the factor analysis. The paper highlights that cultural differences in the manifestation of social anxiety-related behaviors could account for these discrepancies (Heinrichs et al., 2006). Although researchers across different countries have recognized the applicability of the three dimensions of the model to their specific cultural contexts and social media usage patterns, the insights provided by Raimondi et al. (2024) are particularly enlightening for our and other studies involving a three-factor scale. Their findings also pave the way for future validation and application of the SAS-SMU in various countries.

### 6.2 The application of the revised scale: social anxiety of WeChat users in China

The pervasive integration of mobile social media into adults’ lives has transformed social interactions, fostering constant connectivity and accessibility. This heightened engagement in social activities has precipitated a corresponding increase in social anxiety, characterized by its multifaceted and frequent nature. Findings from this study, conducted among WeChat users, indicate elevated levels of overall social anxiety (M > 3). All sub-dimensions of the Social Anxiety Scale exhibited heightened anxiety, with interaction anxiety, a construct present in both offline and online contexts (M = 3.41), manifesting at lower levels than the novel dimensions of social anxiety emerging on mobile social media (MSCA = 3.66, MPCA = 3.94). These results suggest that the unique affordances of mobile media technologies intensify social anxiety among users. Contrary to previous research indicating higher levels of social anxiety among females (Asher et al., 2017; Furmark, 1999), this study found a modest yet significant gender difference, with males exhibiting slightly higher social anxiety than females (M_male_ = 3.69, M_female_ = 3.59).

The present study revealed a notable age difference among mobile social media users, While Erliksson et al. (2020) found no significant age effect on social anxiety. Young adults aged 18–35 exhibited significantly higher levels of social anxiety (M = 3.55–3.80, *p* = 0.001) compared to middle-aged adults aged 36–55 (M = 3.20–3.45). These findings align with previous research on face-to-face communication, which has consistently reported a decline in social anxiety with increasing age (Offord et al., 1996; Jiang, 2018).

Social anxiety, a negative psychological state characterized by distress in social interactions, has emerged as a prevalent concern in the era of mobile social media. The convergence of sociocultural factors and technological advancements has contributed to the intensification of this phenomenon. The ubiquitous and instantaneous nature of mobile social media has transformed individuals into “*media persons*,” (Liu 2015) perpetually immersed in a state of heightened anxiety, characterized by uncertainty, and feelings of inadequacy. Future research should delve deeper into the underlying mechanisms driving social anxiety among mobile social media users, examining its impact on various life domains, and scrutinizing the role of constant connectivity in exacerbating this condition.

## 7 Limitations

This study relied on self-reported data collected via online questionnaires, a methodology susceptible to social desirability bias. Future research could employ physiological measures (e.g., galvanic skin response, electroencephalogram) to enhance data validity. The SAS-SMU, adapted from face-to-face interaction scales (IAS, LSAS), served as the primary measure of social anxiety. While providing valuable insights, the scale may not fully capture the unique manifestations of social anxiety within the Chinese mobile social media context. Developing culturally specific measurement items is recommended for future research. This study focused on the prevalence of social anxiety among WeChat users across demographic groups, but did not delve into the causal relationship between social media use and social anxiety. Future research should investigate the underlying mechanisms linking media use, psychological factors (e.g., problematic social media use, social media fatigue), and social anxiety.

## Data Availability

The raw data supporting the conclusions of this article will be made available by the authors, without undue reservation.
